# Assessing Surgical Extent in Endoscopic Sinus Surgery: A Scoping Review of Scoring Systems

**DOI:** 10.1002/alr.70191

**Published:** 2026-06-01

**Authors:** Alexander Lein, Marie T. Ehrgott, Linda Liu, Archana Jaiswal, Rajiv K. Bhalla, Noel Ayoub, Faris F. Brkic, David T. Liu

**Affiliations:** ^1^ Department of Otolaryngology Head and Neck Surgery Medical University of Vienna Vienna Austria; ^2^ Department of Otorhinolaryngology Manchester University NHS Foundation Trust Manchester UK; ^3^ Departments of Otorhinolaryngology Manchester Centre for Clinical Neurosciences Northern Care Alliance NHS Foundation Trust Salford UK; ^4^ Department of Otolaryngology Head and Neck Surgery Stanford University School of Medicine Palo Alto California USA

**Keywords:** biologics, chronic rhinosinusitis, endoscopic sinus surgery, scoping review, scoring systems, surgical completeness

## Abstract

**Background:**

Endoscopic sinus surgery (ESS) varies substantially in the extent of surgical dissection performed, even when described under the same procedural label. In chronic rhinosinusitis, this heterogeneity may influence postoperative outcomes, revision rates, and response to biologic therapies. This review aimed to identify and characterize available instruments for assessing surgical extent after ESS.

**Methods:**

Scoping review following PRISMA‐ScR guidelines with systematic search of PubMed/MEDLINE, Scopus, and Web of Science.

**Results:**

Twenty‐one studies describing 11 instruments were included: spanning computed tomography (CT)‐based scores (Amsterdam Classification of Completeness of Endoscopic Sinus Surgery [ACCESS], Completion of Surgery Index [CoSI], Sinus Surgery Completeness Score [SSCS], and Residual Ethmoid Cell [REC] score), intraoperative classifications (Lamella–Ostium–Extent–Mucosa [LOEM], complete vs. targeted, and Japanese Rhinologic Society [JRS]), radiologic–surgical concordance metrics, and study‐specific tools. A key conceptual distinction emerged between surgical extent (the procedure performed) and surgical completeness (the anatomical result achieved). CT‐based instruments primarily assess completeness, whereas intraoperative classifications capture extent. Because similar procedures may yield different anatomical results, these dimensions are not interchangeable. Among CT‐based instruments, CoSI demonstrated the most consistent outcome associations, with incomplete prior surgery predicting greater benefit from revision ESS. ACCESS showed preliminary utility in biologic response prediction. LOEM is the only intraoperative system with reported outcome associations, although evidence is limited to a single group. Overall, evidence across instruments remains limited and predominantly retrospective.

**Conclusions:**

CT‐based and intraoperative instruments capture different dimensions of prior surgery and should be selected according to the clinical or research question. Notably, structured scoring consistently reveals that many patients meeting guideline criteria for prior surgery have varying anatomical dissection. Given the association between surgical extent, postoperative outcomes, and biologic therapy response, its standardized assessment warrants evaluation for integration into clinical decision making. However, prospective validation is urgently needed.

## 1 Introduction

Endoscopic sinus surgery (ESS) is the defining surgical procedure of modern rhinology. Since its introduction in the late 1970s to early 1980s [1, 2], continuous advances in visualization, instrumentation, and image‐guided navigation have allowed complex ESS procedures to be performed safely and reproducibly [3].

Despite this evolution, ESS is not a uniform intervention, even when performed under the same label. The term encompasses procedures that differ substantially in anatomical extent, ranging from a limited uncinectomy to a complete bilateral ethmoidectomy with sphenoidotomy [4, 5]. In this context, no universally accepted definition of what constitutes “complete surgery” exists [4, 6, 7, 8]. Multiple studies show that the number of prior procedures does not reflect the adequacy of anatomical dissection [7, 9, 10]. Operative reports and procedural codes (such as current procedural terminology [CPT]) document the intervention performed but do not capture the anatomical result achieved, and may therefore overestimate the completeness of prior surgery. Consequently, two patients with identical surgical histories on paper may represent fundamentally different starting points for further treatment. Moreover, the extent of surgical dissection represents more than a purely technical variable. Retrospective cohort studies suggest that more complete surgery is associated with better postoperative disease control and lower revision rates [6, 7, 8, 9, 10].

The relevance of this problem has grown with the emergence of biologic therapies for chronic rhinosinusitis with nasal polyps (CRSwNPs). Current EPOS2020 and the EPOS/EUFOREA 2023 guidelines require “appropriate prior sinus surgery” as a precondition for biologic eligibility [11, 12]. The 2025 AAO‐HNSF Clinical Practice Guideline on Adult Sinusitis similarly positions biologics for CRSwNP as an option in patients with persistent disease despite prior medical or surgical therapy, without specifying what constitutes adequate surgical management [13]. As a result, patients who have undergone a limited medial antrostomy and those who have undergone a bilateral ESS with removal of all the bony lamellas both fulfill the criterion of “previous surgery.” The distinction has potentially important clinical implications: retrospective data of patients treated with biologics indicate that the extent of prior surgery may influence therapy response, with extended surgical dissection associated with greater improvement in patient‐reported outcomes and more favorable response rates [14, 15, 16, 17]. In an era where biologic therapies carry substantial long‐term costs, the decision between revision surgery and biologics has direct health‐economic implications [18, 19]. The absence of a validated framework for surgical completeness therefore represents a timely gap to address.

Several systems have been proposed to quantify surgical extent in ESS. These systems differ in conceptual framework, anatomical focus, and validation status. No review has systematically mapped their methodological foundations or clinical applications. This review therefore aims to identify and characterize existing instruments, and to evaluate their applicability at the interface of revision surgery and biologic therapy decision making.

## 2 Methods

### 2.1 Study Design and Reporting Framework

This scoping review was conducted in accordance with PRISMA‐ScR guidelines. The review question was formulated using the PCC framework: patients undergoing ESS (primary or revision), any scoring or classification system assessing surgical extent or completeness, and clinical or research settings involving surgical standardization or outcome evaluation.

A systematic search of PubMed/MEDLINE, Scopus, and Web of Science was conducted for all records from inception to February 22, 2026, combining terms for ESS, scoring systems, and surgical extent using Boolean operators and MeSH terms (full search strings in Appendix). Reference lists of included studies were hand‐searched.

### 2.2 Study Selection and Eligibility

**FIGURE 1:**
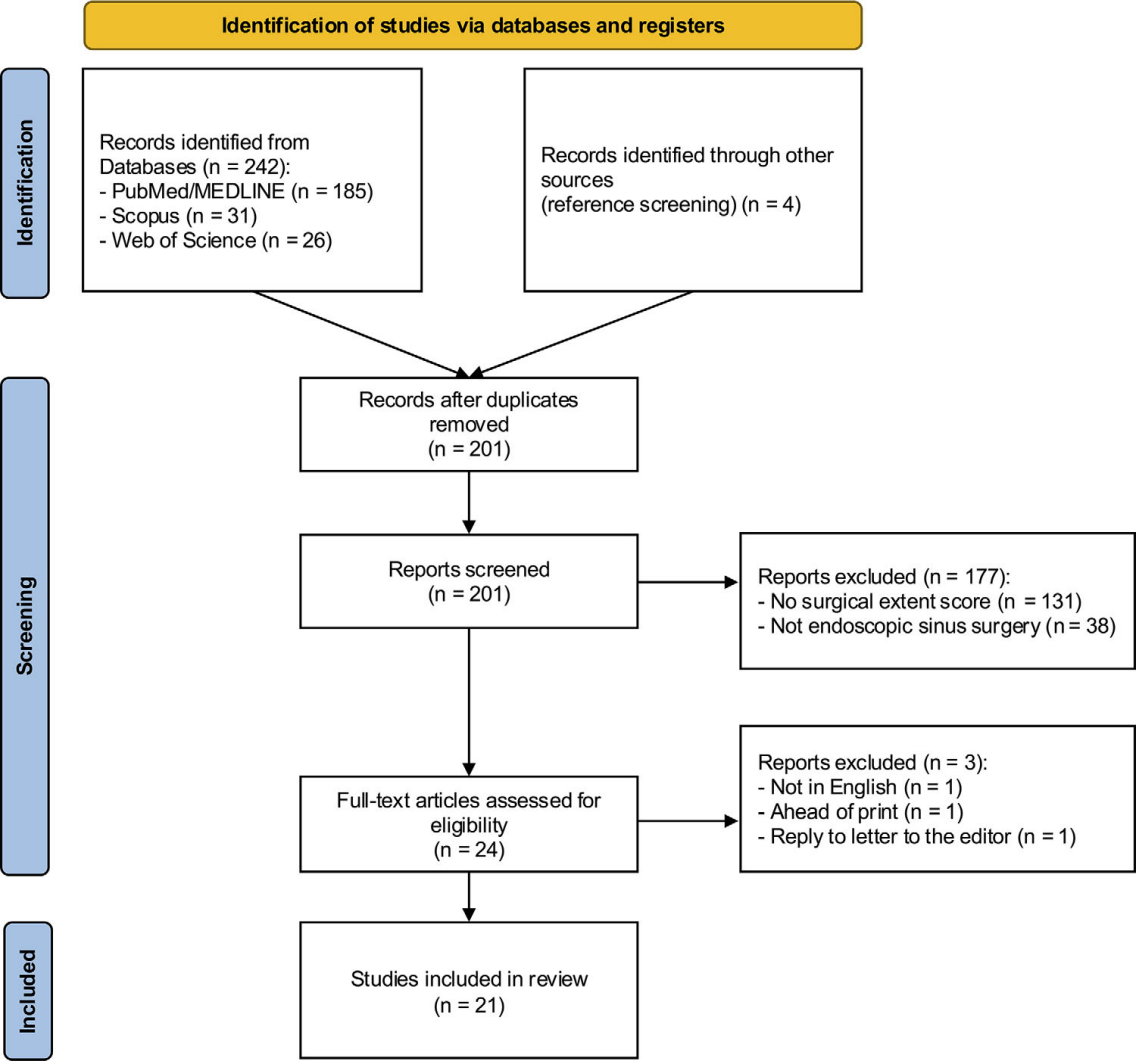
PRISMA flow diagram illustrating the systematic search and study selection process.

Studies were eligible if they described, developed, validated, or applied a system quantifying surgical extent or completeness based on intraoperative documentation, operative reports, or postoperative computed tomography (CT) imaging. No restrictions on study design were applied. Studies were excluded if they addressed symptom scores without reference to surgical extent, perioperative parameters only, non‐endoscopic procedures, or animal and cadaveric studies. Only full‐text English publications were included. Records were screened in Rayyan [20] following duplicate removal, using a two‐stage process of title/abstract and full‐text review (PRISMA‐ScR flow diagram, Figure 1). Records were screened independently by two reviewers (A.L. and M.E.), with disagreements resolved by consensus or, if necessary, by a third reviewer (D.T.L.). Of 242 identified records, 199 were screened after deduplication. Twenty‐four proceeded to full‐text review and 21 were included.

### 2.3 Data Extraction and Synthesis

Data were extracted using a predefined standardized table capturing author, year, country, study design, sample size, scoring system characteristics, data basis, anatomical regions evaluated, scoring scale, validation status, reliability testing, and outcome associations. Instruments were classified into four categories based on data basis and conceptual purpose: CT‐based completeness scores, intraoperative operative extent scores and classifications, radiologic‒surgical concordance metrics and study‐specific instruments.

## 3 Results

### 3.1 Study Overview

A total of 21 studies met the inclusion criteria (Table 1). Studies were published between 2012 and 2026, predominantly from tertiary rhinology centers, and employed retrospective cohort or methodological validation designs. No randomized controlled trials were identified. The identified instruments differed considerably in conceptual framework, anatomical focus, scale structure, and intended application. Overall, the studies described 11 distinct instruments, which are categorized and summarized in Table 2.

**TABLE 1:** Summary of included studies on surgical scoring systems in endoscopic sinus surgery (ESS).

Author, year	Score/system	Study design	Population	*N*	Follow‐up	Primary outcome	Reliability	Key findings	Limitations
Reitsma et al., 2020 [21]	ACCESS	Methodological validation	Mixed sinonasal pathology (CRSwNP/non‐CRS); 6 expert rhinologists; Amsterdam UMC	40 CTs	N/A	Interrater reliability (ICC)	ICC 0.977 (95% CI: 0.964–0.986); superior to LM in low‐opacification subgroup (0.985 vs. 0.816)	First ACCESS validation; ICC 0.977, comparable to LM; sensitive to surgically induced changes (pre/postoperative pairs, *n* = 7); no outcome correlation	Non‐random selection; expert raters only; no outcome data
Schalek et al., 2022 [22]	ACCESS	Retrospective cohort	Revision CRSwNP; asthma 75.5%, N‐ERD 37.8%; single center (Prague)	49	6 months	ACCESS/LM–SNOT‐22 correlation	Two independent rhinologists; no formal ICC	No correlation: ACCESS versus SNOT‐22 pre‐ or postoperative. No correlation LM versus SNOT‐22. LM and ACCESS measure different radiological aspects (*r* = 0.075, *p* = 0.605). Preoperative SNOT‐22 predicted postoperative improvement (*r* = 0.67, *p* < 0.001)	Small sample; single center; no formal ICC; short follow‐up
Alicandri‐Ciufelli et al., 2024 [16]	ACCESS	Multicenter retrospective cohort	Severe CRSwNP on dupilumab, ≥1 prior ESS; asthma 75.8%, N‐ERD 29.6%; 4 Italian centers	145	12 months	ACCESS—dupilumab outcome association	Not reported	Mean ACCESS 8.48; lower ACCESS → better NPS (T1–T3) and SNOT‐22 (T1 only); better VAS obstruction/rhinorrhea (T1, T3). Inverse: higher ACCESS → better VAS smell recovery (T1–T4). Sniffin' sticks: NS	Retrospective; no control group; weak correlations (*r* ^2^ < 5%); high T3–T4 dropout
Pace et al., 2025 [15]	ACCESS	Retrospective cohort	Refractory CRSwNP (type‐2, eosinophilic) on dupilumab, ≥1 prior ESS; single center (Milan, Humanitas)	55	3 and 12 months	ACCESS prediction of dupilumab response	Two independent rhinologists; third for disagreements; references Reitsma 2020	ACCESS only independent predictor of dupilumab response at 12 months (OR = 0.81, *p* = 0.031); AUC = 0.83; cutoff ACCESS ≤13.5 (sens. 82.5%, spec. 70%). No predictor at 3 months	Small sample; single center; no external validation; retrospective
Fuksa et al., 2025 [7]	ACCESS	Retrospective cohort	CRSwNP, revision ESS; prior ‘full house ESS’ declared; asthma 76%, AERD 19%; single center (Prague)	54	≥18 months	ACCESS—disease control/biologic need	Not reported	83% achieved disease control post‐revision ESS. Mean ACCESS: controlled 10.1 ± 0.8 versus uncontrolled 3.7 ± 1.7 (*p* < 0.05). Low ACCESS predicts need for biologics. No correlation between prior surgery count and ACCESS	Retrospective; small sample; single center; no formal reliability
Lazzeroni et al., 2026 [10]	ACCESS	Multicenter retrospective cohort	Diffuse CRS, ≥1 prior ESS; primary 92%, type‐2 87%; asthma 54%; 6 international tertiary centers	114	N/A	Extent of prior ESS (ACCESS distribution)	Not reported	Median ACCESS 12 (IQR 7–17). No Spearman correlation with prior surgery count (*p* = 0.218). Multivariate: each additional surgery associated with ACCESS −1.273 (*p* = 0.025). Frontal/sphenoid most often untouched (63%/55%). Large inter‐center heterogeneity	Retrospective; non‐consecutive; no outcome data; small per‐center sample
Workman et al., 2025 [4]	CoSI	Development and prospective validation; single center (UPenn)	CRSwNP (primary/revision); asthma 75%, AERD 24%, Draf III 46%; University of Pennsylvania	50 (dev.) + 100 (val.)	24 months	CoSI prediction of SNOT‐22 improvement	Mean absolute rater difference 4.3 ± 5.9 pts; no formal ICC/kappa	CoSI 0–100; 70‐pt threshold derived by piecewise regression. Validation: CoSI <70 → SNOT‐22 improvement 28.1 ± 21.7 versus ≥70 → 14.1 ± 12.1 (*p* = 0.029); CoSI‐independent predictor (*p* < 0.01). All postoperative CoSI ≥70	Single center; cutoff derived and validated in same cohort; no formal ICC
Yu et al., 2025 [14]	CoSI	Retrospective cohort	CRSwNP with asthma (AERD 65.6%), revision ESS; single center; University of Pennsylvania	70 (CoSI analyzed)	24 months	CoSI prediction of revision surgery outcomes	≥2 blinded rhinologists; no formal ICC/kappa	CoSI <70 → SNOT‐22 improvement 30.8 ± 20.5 versus ≥70 → 20.4 ± 14.9 (*p* = 0.042); significant at 12 months (*p* = 0.007); independent of biologic use. Uncontrolled asthma subgroup: CoSI <70 → ACT improvement 3.9 ± 2.2 versus 0.88 ± 1.4 (*p* = 0.029)	Single center; retrospective; AERD >50% limits generalizability
Gupta et al., 2025 [23]	SSCS	Development and reliability study	CRSwNP, prior ESS; 2 UK tertiary centers	41	N/A	Interrater reliability (Fleiss *κ*)	Fleiss *κ* = 0.857 (strong); completion time 2.7 min	Mean SSCS 7.40/24; no patient achieved maximum. Frontal/sphenoid undissected in 89%. Weak, NS correlations with LM and SNOT‐22	Small sample; no outcome validation; 2 centers only
Okushi et al., 2012 [24]	REC score	Retrospective cohort	Primary CRS (no revision); single center (Japan)	138	CT ≥6 months post‐ESS	REC association with CRS recurrence	Single blinded rater; no interrater reliability	REC = 0 in 25.4% only. Superior‒anterior ethmoid most incomplete (51.8%). Satisfactory CT outcome (IR ≥ 40%): 70.3%. Poor outcome predictors: eosinophilia ≥9.5% (OR 4.47), asthma (OR 3.09), REC ≥4 (OR 2.48)	Single center; primary CRS only; no interrater reliability; CT outcome only
Levin et al., 2023 [34]	CT surgical completeness score	Retrospective CT reliability study	AERD with prior ESS; 4 blinded rhinologists; single center (Toronto)	61	N/A	Interrater reliability (Fleiss *κ*)	Fleiss *κ* per sinus = 0.418 (moderate); total score *κ* = 0.121 (slight)	Score 0–14 (binary, 7 structures/side). Mean 6.7/14. Frontal adequately opened in ∼22%. Adapted from LM operative component; not purpose‐built; no outcome data	AERD‐specific; poor total‐score reliability; no outcome data
Sánchez‐Gómez et al., 2025a [25]	LOEM	Letter to the Editor (classification introduction)	Classification development; no patient cohort	N/A	N/A	Development of ESS extent classification	Preliminary; details in companion paper (2025b)	LOEM framework (L/O/E/M axes); 4 surgical types (type 1 functional limited → type 4 radical regenerative); codes 5 established ESS techniques; web‐based app (loem.netlify.app)	No outcome data; no quantitative validation; single‐group authorship
Sánchez‐Gómez et al., 2025b [26]	LOEM	Development and Delphi pilot validation	11 ESS video cases; 7 expert rhinologists; 2 Delphi rounds; primary ESS only	11 cases	N/A; 6‐month test–retest	Interrater reliability (*κ*)	Round 1, *κ* = 0.77; Round 2, *κ* = 0.81. Test‒retest *κ* = 0.82. O item test‒retest *κ* = −0.05 (NS)	Overall *κ* improved round 1 → 2 after M‐domain clarification. *L*, *κ* = 1.00; M, *κ* = 0.79. O item unreliable at test‒retest (*κ* = −0.05). Four‐type classification confirmed; web app provided	Expert raters only; video‐based; small case set; no outcome data; primary ESS only
Martin‐Jimenez et al., 2025 [6]	LOEM	Retrospective cohort	CRS (CRSwNP predominant); LOEM types t1–t4; single center (Seville)	305	2 years	LOEM prediction of surgical outcomes	Not reported	t4 SNOT‐22 improvement 50.0 ± 24.1 versus t1/t2 (*p* = 0.003). Adjusted *β* = 7.67 (95% CI: 3.71–11.64, *p* < 0.001). Responder OR t4 = 14.6 (*p* = 0.042); super‐responder OR t4 = 8.49 (*p* = 0.036). Prior ESS: negative predictor (OR = 0.26)	Retrospective; single center; strong confounding by indication; small t4 group (*n* = 30)
Clari‐Comes et al., 2026 [8]	LOEM	Retrospective cohort	CRSwNP, no biologics; LOEM types t1–t4; single center (Seville)	172	3 years	Therapeutic escalation rates by LOEM type	Not reported	t3–t4 versus t1–t2: OR 0.26 (95% CI: 0.08–0.83, *p* = 0.014) for disease escalation; HR 0.46 (Cox, *p* = 0.017). Log‐rank *p* = 0.009. Escalated patients: SNOT‐22 43.8 ± 19.4 versus 18.4 ± 3.7 (*p* < 0.001)	Retrospective; single center; escalation defined endoscopically only; single‐group authorship
González‐García et al., 2025 [27]	LOEM	Systematic review and meta‐analysis	13 studies, predominantly CRSwNP; LOEM retrospectively reclassified in 12/13	2024	Variable (4–6 weeks to 60 months)	QoL and recurrence by LOEM type	Not applicable (review)	Limited versus extended: revision RR 1.76 (95% CI: 1.30–2.38, *p* = 0.0003). SNOT‐22 MD 10.82 (NS after HKSJ correction). VAS MD 2.52 (*p* < 0.00001). LOEM 1/2 versus 3: revision RR 2.77 (*p* = 0.04); LOEM 2/3 versus 4: SNOT‐22 MD 16.9	Retrospective LOEM reclassification; GRADE low–very low; high heterogeneity (*I* ^2^ = 97%, SNOT‐22); single‐group authorship
DeConde et al., 2015 [28]	Complete versus targeted	Prospective multicenter observational cohort	Mixed CRS (CRSwNP/CRSsNP); 4 North American academic centers	311	Mean 13.0 ± 5.5 months	Extent–outcome association (SNOT‐22, B‐SIT)	Not applicable	Complete ESS → greater absolute SNOT‐22 improvement (28.1 vs. 21.9, *p* = 0.011) and B‐SIT (0.8 vs. 0.2, *p* = 0.005). Adjusted *β* = −5.9 (*p* = 0.016). Relative improvement NS	Confounding by indication; non‐randomized; heterogeneous targeted group
Kanai et al., 2017 [30]	JRS ESS classification	Retrospective cohort	CRS (CRSwNP and CRSsNP); single center, single surgeon (Japan)	122	3–6 months	ESS extent–perioperative outcomes association	Not reported	Higher ESS type → longer operative time (*r* = 0.652, *p* < 0.001) and blood loss (*r* = 0.280, *p* = 0.007). Postop improvement in CT, olfaction, nasal resistance. Type III versus IV: NS. Nationally reimbursed since 2014	Single center; single surgeon; no reliability; Japan specific; no PROMs
Öztürk and Bozkurt, 2020 [35]	Surgical extent score (ad hoc)	Retrospective cohort	Barosinusitis (scuba divers; RABS, *n* = 11; CBS, *n* = 14); single center (Istanbul); single surgeon	25	Min. 6 months	Extent–disease group comparison	Not reported	CBS scored higher extent (8.7 ± 2.4 vs. 5.6 ± 2.2, *p* < 0.05). Both groups improved in SNOT‐22 and DRQ; RABS improved more than CBS (*p* < 0.05). Two of 14 CBS patients required revision	Very small sample; barosinusitis specific; single center; no reliability
Ayoub et al., 2020 [31]	Concordance score	Retrospective cohort	CRS (primary/revision); single center (Stanford, USA)	247	6 and 24 months	Extent–CT concordance versus outcomes	Not reported	All groups improved at 6 months (mean 22.0‐pt SNOT‐22, NS between groups). At 24 months: fully concordant (score = 0) → 12.5‐pt greater improvement versus most extensive group (*p* = 0.003). Revision rates NS	No reliability analysis; 34% loss to follow‐up at 24 months; single center
Rohde et al., 2024 [33]	LM ratio	Multicenter observational cohort	CRS (primary/revision); 7 North American centers	828	N/A	Surgery–CT extent ratio (descriptive)	Not reported	Mean LM ratio 1.61; 13% below 1.0 (operated more than diseased). CRSwNP versus CRSsNP: 2.26 versus 1.78 (*p* < 0.001). Primary versus revision: NS. Self‐reflection tool, not a clinical decision instrument	No outcome data; no reliability; cross‐sectional; self‐assessment only

Abbreviations: ACCESS = Amsterdam Classification of Completeness of Endoscopic Sinus Surgery; ACT = asthma control test; AERD = aspirin‐exacerbated respiratory disease; AUC = area under the curve; B‐SIT = brief smell identification test; CoSI = completion of surgery index; CRS = chronic rhinosinusitis; CRSwNP/CRSsNP = CRS with/without nasal polyps; ICC = intraclass correlation coefficient; JRS = Japanese Rhinologic Society; LM = Lund‒Mackay; LOEM = Lamella–Ostium–Extent–Mucosa; N‐ERD = NSAID‐exacerbated respiratory disease; NPS = nasal polyp score; NS = not significant; OR = odds ratio; REC = residual ethmoid cell; SNOT‐22 = Sinonasal Outcome Test‐22; SSCS = sinus surgery completeness score; VAS = visual analog scale.

**TABLE 2:** Characteristics of identified surgical scoring instruments.

Score/system	Type	First description	Objective	Data basis	Anatomical domains	Items (*N*)	Scale/range	Score direction
**CT‐based completeness scores**								
ACCESS	CT‐based completeness score	Reitsma et al., 2020 [21]	Quantify completeness of prior ESS based on functional sinus opening	Postoperative CT (bony boundaries)	Frontal, ant. ethmoid, post. ethmoid, sphenoid, maxillary, OMC (bilateral)	12 (6/side)	0–24 (0–2/site)	Higher = less complete surgery
CoSI	CT‐based completeness score	Workman et al., 2025 [4]	Quantify completeness of prior ESS; provide actionable decision threshold	Postoperative CT (bony anatomy)	Maxillary, ethmoid, sphenoid, frontal; Draf III/middle turbinate (bilateral)	10 subsites	0–100 (0–10/subsite)	Higher = more complete surgery (cutoff ≥70 = adequate)
SSCS	CT‐based completeness score	Gupta et al., 2025 [23]	Quantify completeness of prior ESS using uncinate as key anatomical landmark	Postoperative CT (bony landmarks)	Uncinate, maxillary, ant. ethmoid, post. ethmoid, sphenoid, frontal (bilateral)	12 (6/side)	0–24 (0–2/site)	Higher = more complete surgery
REC score	CT‐based completeness score	Okushi et al., 2012 [24]	Quantify residual ethmoid cells as proxy for surgical completeness	Postoperative CT (vs. preoperative CT)	Sup.‐ant. ethmoid, inf.‐ant. ethmoid, post. ethmoid (bilateral)	6 regions	0–18 (0–3/region)	Higher = more residual disease (worse completeness)
**Intraoperative classification systems**								
LOEM	Intraoperative classification system	Sánchez‐Gómez et al., 2025 [25]	Classify intraoperative ESS extent including mucosal management strategy	Intraoperative surgical coding by surgeon	All paranasal sinuses; lamellae; ostia; mucosal management (L, O, E, M domains)	4 domains	4 categories (t1–t4)	Higher type = more extensive surgery (t4 = radical regenerative)
JRS ESS classification	Intraoperative classification system	Kanai et al., 2017 [30]	Standardize operative ESS extent within national reimbursement framework	Intraoperative procedural documentation	All paranasal sinuses; extended procedures	5 procedure types	Type I–V; ESS score 1–10	Higher = more extensive surgery
Complete versus targeted classification	Intraoperative classification system	DeConde et al., 2015 [28]	Classify surgical extent as complete or targeted ESS	Operative procedure codes (CPT)	Maxillary, ant./post. ethmoid, sphenoid, frontal	4 sinus groups	Binary (complete/targeted)	N/A (categorical)
**Radiologic‒surgical concordance metrics**								
Concordance score	Radiologic‒surgical concordance metric	Ayoub et al., 2020 [31]	Assess concordance between surgical extent and radiographic disease burden	Preoperative CT (LM score) + operative report	Maxillary, ant./post. ethmoid, sphenoid, frontal (bilateral)	10 subsites	−10 to +10	+10 = over‐operated; −10 = under‐operated; 0 = concordant
LM ratio	Radiologic‒surgical concordance metric	Rohde et al., 2024 [33]	Quantify surgical extent relative to radiographic disease burden; surgeon self‐reflection tool	Preoperative CT (LM score) + operative report	Maxillary, ant./post. ethmoid, sphenoid, frontal (bilateral)	10 sinus regions	Continuous ratio (mean 1.61)	Higher = more extensive surgery relative to disease burden
**Study‐specific instruments**								
CT surgical completeness score	Study‐specific derived instrument	Levin et al., 2023 [34]	Quantify completeness of prior ESS in AERD‐specific context; adapted from LM operative component	Postoperative CT scans	Middle turbinate, uncinectomy, frontal, maxillary, ant./post. ethmoid, sphenoid (bilateral)	14 (7/side)	0–14 (0 or 1/structure)	Higher = more complete surgery
FESS score	Study‐specific derived instrument	Öztürk and Bozkurt, 2020 [35]	Quantify surgical extent in barosinusitis context (landmark count)	Intraoperative surgical documentation	Maxillary, ant./post. ethmoid, frontal, sphenoid, frontoethmoidal recess (bilateral)	6 landmark areas	1–12	Higher = more extensive surgery

Abbreviations: ACCESS, Amsterdam Classification of Completeness of Endoscopic Sinus Surgery; AERD, aspirin‐exacerbated respiratory disease; ant., anterior; CoSI, completion of surgery index; CPT, current procedural terminology; ESS, endoscopic sinus surgery; inf., inferior; JRS, Japanese Rhinologic Society; LM, Lund‒Mackay; LOEM, Lamella–Ostium–Extent–Mucosa; OMC, ostiomeatal complex; post., posterior; REC, residual ethmoid cell; SSCS, sinus surgery completeness score; sup., superior.

### 3.2 CT‐Based Completeness Scores

#### 3.2.1 Amsterdam Classification of Completeness of Endoscopic Sinus Surgery (ACCESS)

ACCESS, introduced in 2020, is a CT‐based instrument assessing surgical completeness after ESS [21]. The scoring system evaluates six bilateral anatomical regions on a 0‒2 scale (0 = functionally opened, 1 = addressed but inadequate, and 2 = unoperated), with the ostiomeatal complex scored as 0 or 2 only, yielding a total of 0‒24 where higher scores reflect less complete surgery.

The original validation study demonstrated excellent interrater reliability (ICC 0.977) across six rhinologists assessing 40 CT scans, holding across diagnostic subgroups and varying radiologic disease burdens [21]. Subsequent studies applied ACCESS in tertiary rhinology populations, revision surgery cohorts, and real‐world biologic treatment settings [7, 10, 15, 16, 22]. In one monocentric retrospective cohort, preoperative ACCESS score independently predicted favorable dupilumab response at 12 months, with an area under the curve (AUC) of 0.83 [15]. Across studies, many patients referred to tertiary care showed incomplete prior surgery despite meeting criteria for previous ESS [10, 16, 17].

#### 3.2.2 Completion of Surgery Index (CoSI)

CoSI assesses surgical completeness by scoring the presence or absence of bony partitions on CT imaging [4]. It evaluates the bony opening of the maxillary, ethmoid, sphenoid, and frontal sinuses on a three‐tiered scale of 0, 5, or 10 points per side, with additional points for partial middle turbinectomy and Draf III frontal sinusotomy, yielding a total range of 0‒100.

A threshold of 70, derived from piecewise regression and validated in an independent cohort, distinguishes incomplete from adequate prior surgery [4]. Patients with CoSI <70 showed greater Sinonasal Outcome Test‐22 (SNOT‐22) improvement after revision ESS compared to those with more complete prior surgery (28.1 vs. 14.1 points, *p* = 0.029), independent of asthma and biologic use [4]. This finding was replicated in a cohort of CRSwNP patients with asthma, with greater improvements in both SNOT‐22 (30.8 vs. 20.4, *p* = 0.042) and Asthma Control Test scores (3.9 vs. 0.88, *p* = 0.029) [14]. Correlation with Lund–Mackay (LM) scores was weak and non‐significant.

#### 3.2.3 Sinus Surgery Completeness Score (SSCS)

The SSCS, introduced in 2025, assesses surgical completeness on postoperative CT by evaluating six bilateral anatomical regions on a 0–2 scale, yielding a total score of 0–24, with higher scores indicating more complete surgery [23]. Unlike ACCESS, it includes the uncinate process as a separate subunit rather than using the ostiomeatal complex as a proxy.

In a cohort of 41 CRSwNP revision patients across two UK tertiary centers, SSCS demonstrated strong interrater reliability (Fleiss’ *κ* = 0.857, completable in under 3 min). Mean SSCS was only 7.40/24, with the frontal and sphenoid sinuses were completely undissected in 89% of cases [23]. Correlations with LM and SNOT‐22 were not statistically significant [23].

#### 3.2.4 Residual Ethmoid Cell (REC) Score

The REC score quantifies residual laminae and cells across six bilateral ethmoid subregions on postoperative CT, yielding a total score of 0‒18 [24]. In a cohort of 138 primary chronic rhinosinusitis (CRS) patients, an REC score ≥4 independently predicted poor postoperative outcome (odds ratio [OR] 2.477) [24]. The superior‒anterior ethmoid was the most frequently incompletely cleared region [24]. No interrater reliability analysis was reported, and the instrument has not been evaluated beyond the original study.

### 3.3 Intraoperative Classification Systems

#### 3.3.1 Lamella–Ostium–Extent–Mucosa (LOEM) Classification

LOEM is an intraoperative classification system that encodes the surgical procedure performed without reliance on postoperative imaging [25, 26]. The system encodes each procedure using four domains: lamella (L, anteroposterior bone removal extent across four lamellae), ostium (O, sinus ostium enlargement), extension (E, extended approaches beyond standard antrostomy), and mucosa (M, mucosal management strategy ranging from functional preservation to complete removal and regenerative mucoplasty). These domains combine into four surgical type designations: type 1 (functional limited) through type 4 (radical regenerative). Each type is defined by increasing extent of bone removal, ostium enlargement, and mucosal intervention.

Preliminary reliability was established across two Delphi rounds among seven experienced rhinologists evaluating 11 ESS video cases, with overall agreement improving from *κ* = 0.77 to 0.81 and test‒retest reliability of *κ* = 0.82 at 6 months [25].

Clinical data are limited to studies from a single research group. In a cohort of 305 CRS patients, type 4 surgery was the independently associated with greater SNOT‐22 improvement (adjusted OR = 8.5). In a cohort of 172 CRSwNP patients, more extensive surgery (type 3‒4) was associated with significantly lower rates of therapeutic escalation at 3 years (HR = 0.46) [8]. A systematic review applying LOEM retrospectively across 13 studies found that LOEM 4 produced the greatest quality‐of‐life benefit (SNOT‐22 MD +16.92, exceeding the MCID), while LOEM 1/2 was associated more than twice the recurrence risk of LOEM 3 [27].

#### 3.3.2 Complete Versus Targeted Surgical Extent Classification

DeConde et al. classified surgical extent as a binary variable, defining ESS as “complete” (bilateral maxillary antrostomy, total ethmoidectomy, sphenoidotomy, and frontal sinusotomy) or “targeted” (any lesser intervention), based on CPT codes [28]. In a prospective multicenter cohort of 311 CRS patients, complete surgery was independently associated with greater SNOT‐22 improvement after adjustment for asthma, ASA sensitivity, nasal polyposis, and prior surgery (adjusted *β* = −5.9, *p* = 0.016), although the difference did not reach the MCID [28]. No reliability analysis was reported, and the classification has not been applied beyond this cohort.

#### 3.3.3 Japanese Rhinologic Society (JRS) ESS Classification

JRS ESS Classification, introduced in 2013 and evaluated in 2017 [29, 30], provides a five‐tier framework for operative documentation, ranging from type I (ostiomeatal complex only) to type V (extended procedure beyond the sinus wall), with an additive bilateral ESS score of 1‒10 [30]. In a retrospective cohort of 122 patients, higher ESS type correlated with longer operation time (*r* = 0.652, *p* < 0.001) and greater intraoperative blood loss [30]. The classification was incorporated into the Japanese national health insurance reimbursement system. No reliability analysis was reported, and the instrument has not been evaluated outside Japan.

### 3.4 Radiological‒Surgical Concordance Metrics

#### 3.4.1 Concordance Score

Ayoub et al. introduced the concordance score to assess alignment between surgical extent and preoperative radiographic disease burden [31]. Each of five bilateral sinus regions is scored as 0 (surgery concordant with CT findings), +1 (sinus opened despite no radiographic disease), or ‒1 (diseased sinus left unopened), yielding a total score of ‒10 to +10 [31]. In a cohort of 247 CRS patients, the concordance score was not associated with SNOT‐22 improvement at 6 months or revision rates at 24 months [31]. No reliability analysis was reported.

#### 3.4.2 Lund‒Mackay to Operated Sinus Ratio (LM Ratio)

Rohde et al. proposed the LM ratio, calculated by dividing the modified preoperative LM score [33] (excluding the ostiomeatal complex, maximum 20) by the number of surgically addressed sinus regions, yielding a continuous ratio dichotomized at 1.0 [33]. In a cohort of 828 CRS patients, the mean ratio was 1.61, with higher values in CRSwNP than CRS without nasal polyp (CRSsNP) patients (2.26 vs. 1.78, *p* < 0.001) [33]. No outcome association or reliability analysis was reported.

### 3.5 Study‐Specific Extent Instruments

Levin et al. adapted the operative documentation component of the LM system into a CT‐based completeness score evaluating seven anatomical structures per side (uncinectomy, maxillary, anterior ethmoid, posterior ethmoid, sphenoid, frontal, and middle turbinate reduction), yielding a total score of 0–14 [34]. In 61 aspirin‐exacerbated respiratory disease (AERD) patients, interrater agreement was moderate for individual sinuses (mean *κ* = 0.418) but low for total scores (*κ* = 0.121) [33]. No outcome association was reported [34].

Öztürk and Bozkurt described a landmark‐based extent metric in patients with paranasal barosinusitis, calculated by counting bilaterally operated anatomical regions, yielding a score of 1–12 [35]. In a cohort of 25 patients, the chronic barosinusitis group had higher scores than the recurrent acute group (8.7 vs. 5.6, *p* < 0.05), with both groups showing postoperative SNOT‐22 improvement [35]. No reliability analysis was reported.

## 4 Discussion

The extent of prior ESS is increasingly recognized as a clinically relevant variable in the management of refractory CRS, yet no standardized framework exists for its assessment. This scoping review identified 21 studies describing 11 instruments for the assessment of surgical completeness or extent in ESS. Four instruments (ACCESS, CoSI, SSCS, and LOEM) were developed through a formal process with defined scoring rules and preliminary reliability data. The remaining seven represent either context‐specific adaptations, adaptations of existing tools, or approaches not evaluated beyond the originating study. No randomized controlled trials were identified. The available evidence is uniformly retrospective and predominantly single center.

A key finding of this review is that existing instruments capture two closely related but distinct dimensions (Table 3): surgical extent, referring to the procedure performed, and surgical completeness, reflecting the anatomical result achieved. This conceptual distinction is reflected in the design of the respective instruments. CT‐based scores [4, 21, 23, 24] primarily assess surgical completeness by evaluating the postoperative anatomical state of the sinuses, whereas intraoperative classification systems [25, 26, 28, 30] such as LOEM encode surgical extent by describing the procedure performed. As a result, two patients with similar operative labels may differ substantially in anatomical completeness, and conversely, similar anatomical findings may arise from different surgical approaches. Of note, radiologic concordance metrics represent a third category that bridges both dimensions, asking whether the extent of surgery was appropriate relative to the preoperative radiographic disease burden. They are therefore primarily suited for quality assessment purposes.

**TABLE 3:** Taxonomic classification of surgical scoring instruments and their conceptual implications.

Category	Instruments	Data basis	Conceptual question	Clinical implication	Evidence status
CT‐based completeness scores	ACCESS (Reitsma et al., 2020) [21] CoSI (Workman et al., 2025) [4] SSCS (Gupta et al., 2025) [23] REC score (Okushi et al., 2012) [24]	Postoperative CT imaging	How anatomically complete was a prior operation?	Retrospective quality assessment of prior ESS; potential triage tool for revision surgery versus biologic therapy eligibility	ACCESS and CoSI: preliminary outcome associations. SSCS: reliability only. REC score: single center, not replicated.
Intraoperative classification systems	LOEM (Sánchez‐Gómez et al., 2025) [25] JRS ESS Classification (Kanai et al., 2017) [30] Complete versus targeted (DeConde et al., 2015) [28]	Intraoperative documentation by operating surgeon	What type of operation was performed?	Prospective standardization of operative documentation; enables comparability across surgeons and centers; does not assess anatomical result	LOEM: preliminary outcome associations (single group). JRS and DeConde: limited to originating study.
Radiologic‒surgical concordance metrics	Concordance score (Ayoub et al., 2020) [31] LM ratio (Rohde et al., 2024) [33]	Preoperative CT disease burden + operative extent	Was the extent of surgery appropriate relative to radiographic disease burden?	Quality improvement and surgeon self‐reflection; not designed as a clinical decision instrument; no outcome associations established	Descriptive only; neither instrument replicated beyond originating study.

Abbreviations: ACCESS, Amsterdam Classification of Completeness of Endoscopic Sinus Surgery; CoSI, Completion of Surgery Index; CT, computed tomography; ESS, endoscopic sinus surgery; JRS, Japanese Rhinologic Society; LM, Lund‒Mackay; LOEM, Lamella‒Ostium‒Extent‒Mucosa; REC, residual ethmoid cell; SSCS, sinus surgery completeness score.

Even within CT‐based instruments, important conceptual differences exist. ACCESS evaluates whether sinus cavities have been functionally opened [21], thereby incorporating an element of clinical judgement, whereas CoSI focuses exclusively on the presence or absence of bony partitions, prioritizing structural reproducibility [4]. SSCS adopts a third approach by selecting the uncinate process as a discrete anatomical subunit rather than using the ostiomeatal complex as a proxy [23]. These differences illustrate an unresolved question within the field: which anatomical structures most accurately capture surgical intervention and can be reliably assessed on imaging.

The lack of conceptual distinction contributes to heterogeneity in outcome studies and limits comparability across cohorts. No study to date has applied two instruments within the same patient cohort. As a result, it remains unknown whether a patient classified as incomplete by CoSI would also score high on ACCESS, or whether a LOEM type 4 procedure reliably produces a low ACCESS score. A prospective cohort applying at least one CT‐based and one intraoperative instrument simultaneously would allow direct comparison between what operation was performed and how complete the surgical result was and would test whether the conceptual distinction between surgical extent and surgical completeness has measurable clinical consequences.

Among the CT‐based instruments, CoSI currently provides the most consistent outcome‐related data. Its 70‐point threshold, the only scoring threshold in the literature derived and validated across two independent cohorts, identifies patients with incomplete prior surgery who achieve revision outcomes comparable to primary surgical patients [4]. ACCESS has been applied across the broadest range of clinical contexts, including biologic response prediction and treatment escalation, with an AUC of 0.83 for dupilumab response in one cohort [15]. Its outcome associations, however, remain exploratory and single center. LOEM has generated the most mechanistically ambitious hypotheses. However, all supporting evidence originates from a single group, which substantially limits the generalizability of current findings [6, 8, 27]. Taken together, the evidence base is uneven: CoSI is the only instrument with a replicated outcome association, ACCESS has the broadest clinical application but no external validation, and LOEM was assessed the most, but with least methodological independence. No instrument has been prospectively validated as a clinical decision tool.

Emerging evidence suggests that surgical extent influences both revision outcomes and response to biologic therapy. Current approval criteria for all licensed biologics [36, 37, 38, 39, 40], as well as international clinical practice guidelines [11, 12, 13], define prior surgery in binary terms, without accounting for the degree of surgical dissection. Three studies have suggested that the degree of prior surgical dissection, as measured by ACCESS, predicts biologic response and the likelihood of requiring escalation after revision surgery [7, 15, 16]. Pace et al. found ACCESS to be the only independent predictor of dupilumab response at 12 months [15], Alicandri‐Ciufelli et al. reported better early polyp and symptom outcomes in patients with more extensive surgery [16], and Fuksa et al. showed that lower preoperative ACCESS predicted the need for biologic escalation after revision surgery [7]. All three studies are retrospective and single center. Notably, olfactory recovery under dupilumab was worse in more extensively operated patients, a finding that warrants further investigation [16]. Importantly, the CoSI cohorts support this pattern from a complementary perspective: Workman et al. and Yu et al. both demonstrated that incomplete prior surgery predicted greater SNOT‐22 improvement after revision, an effect that held independently after controlling for biologic use [4, 14]. Taken together, these data suggest that not all prior surgery is equal.

The clinical implications are twofold: patients with incomplete prior surgery may benefit from revision first, with CoSI data suggesting their outcomes are comparable to primary surgical patients [4], while those with complete prior surgery who continue to have uncontrolled disease may represent a population in whom earlier biologic initiation should be considered. With remission emerging as a treatment target in CRSwNP [41, 42], standardized assessment of prior surgical extent becomes a research priority. No randomized trial has tested this pathway, and no instrument has been prospectively validated for this purpose.

Beyond methodological differences, the identified scores reveal consistent anatomical patterns in prior ESS. In the SSCS cohort, the frontal and sphenoid sinuses were completely undissected in 89% of revision candidates [23]. Moreover, in the multicenter study by Lazzeroni et al., an ACCESS score of 2 (indicating no prior surgical intervention) was recorded for the frontal sinus in 63% and the sphenoid sinus in 55% of cases [10]. The REC score, similarly identified the superior‒anterior ethmoid as the most frequently incompletely cleared region, with residual cells present in 51.8% of cases [24]. Of note, while studies on the CoSI score did not investigate a specific undissected area, it is very frontal sinus heavy, allowing full scores only with a performed Draf III. Currently, a prior maxillary antrostomy and anterior ethmoidectomy satisfy most guideline definitions of prior surgery yet leaves multiple assessed regions entirely unoperated. The instruments reviewed here make it possible to identify and quantify this discrepancy. Whether patients with such surgical histories should be considered adequately treated prior to biologic initiation remains an open question.

Several important gaps in the literature extend beyond any single instrument. CRSsNP is substantially underrepresented across all included studies. None of the CT‐based scores captures mucosal quality, tissue eosinophilia, or surgical technique factors that may independently determine outcomes. LOEM, conversely, does not interface with CT‐based disease burden. Formal cost‐effectiveness analyses of score‐guided treatment pathways are absent. Prospective multicenter studies applying standardized instruments across diverse surgical teams, patient phenotypes, and healthcare systems are needed before any of these instruments can be considered validated clinical decision‐support tools.

This review has several limitations inherent to scoping review methodology. The included literature was heterogeneous in study design, patient populations, and outcome reporting. Furthermore, several instruments have only been evaluated by the groups that originally developed them. Given the substantial variation in surgical practice patterns across centers and countries, independent validation by non‐developer groups and in multi‐institutional cohorts will be most informative for establishing the generalizability and reliability of existing instruments.

## 5 Conclusion

This scoping review identified different concepts for the assessment of surgical completeness or extent after ESS. CT‐based completeness scores evaluate the anatomical result of prior surgery on postoperative imaging, while intraoperative classification systems describe the operative strategy performed. These methodological differences contribute to heterogeneity in outcome studies and limit comparability across cohorts. Importantly, available studies indicate that many patients meeting criteria for prior ESS demonstrate anatomically variable surgical dissection when evaluated using structured scoring systems. Current evidence suggests that the degree of prior surgical dissection may influence both revision surgery outcomes and response to biologic therapy. However, existing treatment algorithms define prior surgery in binary terms without accounting for surgical extent. Therefore, evaluation of surgical extent warrants to be included in clinical decision making. No instrument has been prospectively validated as a clinical decision‐support tool, and no direct comparisons between scoring systems exist. An ideal future study would be prospective and multicentric, apply at least one CT‐based and one intraoperative instrument to the same cohort, and include both polyp and non‐polyp CRS phenotypes, with structured assessment of patient‐reported outcomes, revision rates, and biologic treatment response. Such a design would clarify whether the conceptual distinction between surgical extent and completeness has measurable clinical consequences, and whether any single instrument is sufficient for clinical decision making.

## Funding

No funding was received for this work.

## Conflicts of Interest

The authors declare no conflicts of interest.

## Supporting information




**Supporting File 1**: Appendix 1 – Search Strategy

**Supporting File 2**: Preferred Reporting Items for Systematic reviews and Meta‐Analyses extension for Scoping Reviews (PRISMA‐ScR) Checklist
