# Supplementary material for: Assessing Surgical Extent in Endoscopic Sinus Surgery: A Scoping Review of Scoring Systems
Source: Int Forum Allergy Rhinol. 2026 Jun 1;16(7):695–715. doi: 10.1002/alr.70191 (PMC13326797; doi:10.1002/alr.70191)
Supplement: Supplementary file 1 — Supporting File 1: Appendix 1 – Search Strategy [file ALR-16-695-s001.docx]

**Appendix 1 – Search Strategy**

A systematic literature search was performed to identify studies describing, developing, validating, or applying scoring systems intended to assess the extent or completeness of endoscopic sinus surgery (ESS). Both controlled vocabulary terms (e.g., MeSH terms in PubMed) and free-text keywords were used. Boolean operators (AND, OR) were applied to combine search terms. Equivalent search strategies were adapted for each database according to their specific syntax requirements. No date restrictions were applied. Only English-language full-text articles were included.

Pubmed: ( "Endoscopic Sinus Surgery"[Mesh] OR "endoscopic sinus surgery" OR "functional endoscopic sinus surgery" OR ESS OR FESS ) AND ( "extent of surgery" OR "surgical extent" OR "extent of endoscopic sinus surgery" OR "completeness of surgery" OR "surgical completeness" OR "classification of surgery" OR "classification of endoscopic sinus surgery" ) AND ( score* OR "scoring system*" OR "surgical score*" OR "operative score*" )

Scopus: TITLE-ABS-KEY ( "endoscopic sinus surgery" OR "functional endoscopic sinus surgery" OR ESS OR FESS ) AND TITLE-ABS-KEY ( SCORE OR "scoring system" OR "surgical score" OR "operative score" ) AND TITLE-ABS-KEY ( "surgical extent" OR "extent of surgery" OR "surgical completeness" OR "completeness of surgery" OR "classification of surgery" )

Web of Science (Core Collection): ("endoscopic sinus surgery" OR "functional endoscopic sinus surgery" OR ESS OR FESS) AND ("surgical extent" OR "extent of surgery" OR "surgical completeness" OR "completeness of surgery" OR "classification of surgery") AND (score OR "scoring system" OR "surgical score" OR "operative score")

Additional Search Methods: In addition to electronic database searches, the reference lists of all included studies were manually screened to identify additional relevant publications not captured by the database search. Through this reference screening process, two additional studies were identified and included in the final review.
